# Conserved and differential transcriptional responses of peroxisome associated pathways to drought, dehydration and ABA

**DOI:** 10.1093/jxb/ery266

**Published:** 2018-07-19

**Authors:** Heba T Ebeed, Sean R Stevenson, Andrew C Cuming, Alison Baker

**Affiliations:** 1 Botany and Microbiology Department, Faculty of Science, Damietta University, Damietta, Egypt; 2 Centre for Plant Sciences, University of Leeds, Leeds, United Kingdom

**Keywords:** ABA, antioxidant, bioinformatics, drought, moss, peroxisome, PEX, *Physcomitrella patens*, *Triticum aestivum*, wheat

## Abstract

Plant peroxisomes are important components of cellular antioxidant networks, dealing with ROS generated by multiple metabolic pathways. Peroxisomes respond to environmental and cellular conditions by changing their size, number, and proteomic content. To investigate the role of peroxisomes in response to drought, dehydration and ABA treatment we took an evolutionary and comparative genomics approach. Colonisation of land required evolution of dehydration tolerance in the absence of subsequent anatomical adaptations. Therefore, the model bryophyte *Physcomitrella patens*, the model dicot *Arabidopsis thaliana* and wheat (*Tricitcum aestivum*), a globally important cereal crop were compared. Three sets of genes namely ‘*PTS1* genes’ (a proxy for genes encoding peroxisome targeted proteins), *PEX* genes (involved in peroxisome biogenesis) and genes involved in plant antioxidant networks were identified in all 3 species and their expression compared under drought (dehydration) and ABA treatment. Genes encoding enzymes of β-oxidation and gluconeogenesis, antioxidant enzymes including catalase and glutathione reductase and *PEX3* and *PEX11* isoforms showed conserved up-regulation, and peroxisome proliferation was induced by ABA in moss. Interestingly, expression of some of these genes differed between drought sensitive and resistant genotypes of wheat in line with measured photosynthetic and biochemical differences. These results point to an underappreciated role for peroxisomes in drought response.

## Teaser

Peroxisome biogenesis, peroxisome β-oxidation and antioxidant enzymes show conserved upregulation in response to ABA and water deficit-inducing treatments, highlighting an overlooked role for peroxisomes in response to drought.

## Introduction

Water deficiency is a severe constraint on crop production world-wide (Boyer, 1982). For example drought regularly limits wheat production in almost 50% of the cropped area. This issue is of increasing concern and is amplified by climate change, population growth and urbanization, impacting on water availability for agriculture and therefore global food security (Godfray *et al.*, 2010). Consequently new insights into the molecular mechanisms of response to drought is an important but challenging goal for improvement of drought tolerant plant varieties (Claeys and Inzé, 2013)

Abscisic Acid (ABA) is a major player in coordinating the adaptation of plants to adverse conditions as well as functioning in many plant developmental processes (Leung and Giraudat, 1998; Fujita *et al.*, 2011; Stevenson *et al.*, 2016). ABA mediates physiological processes such as stomatal closure, osmolyte accumulation, and the synthesis of stress-related proteins, as well as compounds associated with the scavenging of reactive oxygen species that are implicated in desiccation related membrane damage (Ingram and Bartels, 1996; Hoekstra *et al.*, 2001). ABA is required for the induction of genes as a response to dehydration stress (Nakashima *et al.*, 2009). Moreover, exogenous application of ABA induces a number of genes that respond to dehydration and cold stress (Zhu, 2002; Shinozaki *et al.*, 2003; Cuming *et al.*, 2007) However, not all genes that are induced by dehydration and cold stress respond to the exogenous application of ABA (Zhu, 2002; Yamaguchi-Shinozaki and Shinozaki, 2006). This suggests the existence of ABA-independent and ABA-dependent signal transduction pathways that convert the initial stress signal into cellular responses (Zhu, 2002).

The recruitment of ABA to regulate responses to water stress emerged with the evolution of land plants, which are monophyletic in origin, descending from a single successful colonisation of terrestrial habitats by a charophyte algal ancestor *ca*. 470Ma (Delwiche and Cooper, 2015). The conquest of land necessarily required adaptations enabling these ancestral plants to survive the highly variable conditions characteristic of the terrestrial habitats, most notably exposure to ultraviolet radiation, salinity, dehydration and temperature variation. Lacking the anatomic adaptations characteristic of extant tracheophytes, survival of the earliest colonisers must have been cellular and biochemical in nature. A common cellular consequence of these environmental stresses is the formation of reactive oxygen species (ROS). Consequently, possession of antioxidant mechanisms must have ranked highly in the suite of adaptations that supported the transition from aquatic to terrestrial habitats, enabling both ROS signaling and defence against ROS toxicity. Such adaptations remain important today, being widespread and highly conserved in nature among all classes of land plant and central to many environmental stress responses (Mittler *et al.*, 2011; Noctor *et al.*, 2014).

Peroxisomes are both major sources of ROS and sites of important anti-oxidant defences (Noctor *et al.*, 2002). They contain antioxidant molecules such as ascorbate and glutathione, and some antioxidant enzymes, including ascorbate peroxidase, dehydro- and monodehydroascorbate reductase, glutathione reductase and catalase. Changes in activities of these enzymes are regulated by various stress conditions (del Rio *et al*., 1998). Accordingly, peroxisomes have been suggested to play important roles in defence against abiotic and biotic stress in plants (Willekens *et al.*, 1997; del Rio *et al*., 1998). They are involved in lipid mobilization through β-oxidation and the glyoxylate cycle, photorespiration, nitrogen metabolism, synthesis and metabolism of plant hormones (Hu *et al.*, 2012). Peroxisomes import membrane and soluble proteins from the cytosol to maintain and modulate their functions (review (Cross *et al.*, 2016).The biogenesis of peroxisomes requires a group of protein factors referred to as peroxins encoded by *PEX* genes (Distel *et al.*, 1996). Two types of targeting signals have been identified for peroxisomal matrix enzymes: PTS1, a C-terminal tripeptide and PTS2, an N-terminal nonapeptide (Reumann *et al.*, 2016). Peroxisome membrane proteins are inserted post translationally by the action of chaperone/receptor PEX19 and its docking factor PEX3. Some membrane proteins may also be targeted to peroxisomes via the ER in a process that also requires PEX3 (Cross *et al.*, 2016).

Peroxisomes are remarkably dynamic, responding to environmental and cellular cues by alterations in size, number, and proteomic content. As well as importing proteins from the cytosol, peroxisomes proliferate by division in a process dependent upon the PEX11 family (Orth *et al.*, 2007; Kamisugi *et al.*, 2016). Plant peroxisome proliferation has been reported in response to hydrogen peroxide, pathogens or ozone (Morré *et al.*, 1990; Lopez-Huertas *et al.*, 2000; Oksanen *et al.*, 2004), and during senescence (Pastori and Del Rio, 1997).

To investigate the evolving roles of peroxisomes in perception and response to abiotic stress we focused on drought and its consequences: dehydration stress, ABA production and ROS metabolism. We have taken a genome wide cross species approach, utilising information gained from a modern angiosperm and from a bryophyte—the most ancient group of land plants—to compare transcriptional responses of PTS1 targeted peroxisome proteins, antioxidants and *PEX* genes. We benefit from the plethora of genomic resources available for the well characterised angiosperm and bryophyte models, *Arabidopsis thaliana* and *Physcomitrella patens* and extend these studies to the globally preeminent crop species, wheat (*Triticum aestivum*), for which comparable resources are only now being developed (Uauy, 2017). Due to its large hexaploid genome wheat is a much more challenging species to study than haploid *Physcomitrella* and diploid *Arabidopsis thaliana*, therefore we used the rich data and extensive information from these two model species to demonstrate that genes encoding peroxisome targeted proteins are disproportionally upregulated and that upregulation of peroxisomal β-oxidation is a conserved response to drought, dehydration and ABA. Additionally peroxisome biogenesis appears to be upregulated with increased expression of isoforms of *PEX3* and *PEX11* seen in both moss and wheat with clear differences between drought sensitive and drought tolerant cultivars. Interestingly increased expression of glyoxylate cycle enzymes ICL and MS is seen in moss and wheat but not in Arabidopsis.

## Materials and methods

### Compiling Arabidopsis peroxisomal genes and identification of homologs in moss and wheat

Arabidopsis proteins predicted to be targeted to peroxisomes were retrieved from AraPerox 1.2 (Reumann *et al.*, 2007). The antioxidant genes, their description and localization information were compiled manually from The Arabidopsis Information Resource (www.arabidopsis.org). Those Arabidopsis antioxidant enzymes annotated as peroxisomal were used to identify non-peroxisomal isoenzymes and some additional known non-peroxisomal components of the anti-oxidant network from Arabidopsis were also added. This resulted in a list of 51 Arabidopsis proteins, representing 10 families.

To identify homologs for genes encoding PTS1-containing proteins, *PEX* proteins, and antioxidant enzymes (‘*PTS1*, *PEX* and *Antox*’ genes respectively) in *P. patens* and wheat, the whole protein sequence content of *Arabidopsis thaliana* was obtained from TAIR and Arabidopsis proteins were used to search the *Physcomitrella* and wheat genomes at http://phytozome.jgi.doe.gov/ (E-value<1e-10 and <1e-5, respectively) by TBLASTN to identify homologs. Then, all the sequences of unique hits in wheat or moss were used for reciprocal BLASTP search of the Arabidopsis proteome. Due to the large hexaploid wheat genome, wheat homologs for PTS1-containing proteins and antioxidant enzymes were obtained by separate queries using BioMart (http://www.gramene.org/) (Gupta *et al.*, 2016), and the TAIR Arabidopsis dataset. Data were filtered to obtain corresponding homologs in *Triticum aestivum*, then gene stable IDs were converted to corresponding Ensembl gene ID manually by TBlastN analysis of protein sequences against the wheat genome at Phytozome (*E*-value<1e-5) to identify the best blast match for each locus. PredPlantPTS1 (http://ppp.gobics.de/) (Reumann *et al.*, 2012) was used for the prediction of PTS1 signals in moss and wheat homologs of genes of putative PTS1 proteins. All candidate homologs were verified with the help of CDD and Expasy databases (https://www.expasy.org/) (Gasteiger *et al.*, 2003) to confirm the presence of expected conserved domains. All the moss and wheat proteins identified from BLAST searches were accepted only if they contained the corresponding Arabidopsis domains; then multiple sequence alignments were used to confirm the conserved domains of identified sequences. Retrieved sequences in wheat were corrected when a portion of protein was missing due to incorrect gene model prediction. Sequences showing large truncations and that could not be completed by further BLAST searches were excluded.

### Peroxisomal gene expression in moss under ABA, dehydration and mannitol

The gene expression profiles of the *Physcomitrella* peroxisomal (*PTS1*, *PEX*) and *Antox* genes responding to ABA, osmotic- and dehydration- stress were obtained using the RNA-seq data deposited in the Gene Expression Omnibus database under accession number GSE72583 and then to the NCBI Sequence Read Archive (accession number SRP063055; BioProject PRJNA294412) (Stevenson *et al.*, 2016). To assess statistical significance, hypergeometric probabilities were evaluated for the number of genes in the data set of interest (eg. *PTS*1, *PEX* or *Antox*) up-regulated ≥2-fold change (FC) by the experimental treatment compared to the total number of genes up-regulated ≥2 FC in the entire gene set for that treatment. The heatmaps were drawn using the Morpheus software (https://clue.io/morpheus/) (Minguet *et al.*, 2015).

### Peroxisomal gene expression in Arabidopsis in response to ABA treatment

Arabidopsis RNAseq expression data were downloaded from the Gene Expression Omnibus (GEO, http://www.ncbi.nlm.nih.gov/geo/) (GEO accession number: GSE65739 and SRA accession number: SRP053346) and 4 samples, two biological replicates of 10-day-old Arabidopsis seedlings mock treated (GSM1603932 GSM1603936) or treated with 50 μM ABA, (GSM1603933, , GSM1603937) (Weng *et al.*, 2016) were selected to study expression of our candidate genes. Processed data files were downloaded.

### Peroxisomal gene expression in *Triticum aestivum* under drought stress

Wheat transcriptome profiling and gene expression data were retrieved from the Gene Expression Omnibus (GEO, http://www.ncbi.nlm.nih.gov/geo/) (GEO accession number: GSE30436) (Kadam *et al.*, 2012). Twelve samples were selected to study expression of candidate genes in two bulked populations of wheat recombinant inbred lines which differed in their susceptibility to drought ‘drought sensitive Bulk’ and ‘drought tolerant Bulk’. The sample accession numbers are as follows: GSM754878, GSM754879, GSM754880, GSM754884, GSM754885, GSM754886, GSM754890, GSM754891, GSM754892, GSM754896, GSM754897, GSM754898, three samples were used as a biological replicate for each treatment. CEL files were downloaded and processed data values for the selected samples were used to calculate FC in tolerant and sensitive genotypes. Probesets corresponding to *PTS1, PEX* and *Antox* genes were searched using an online PLEXdb Blast tool available at Affymetrix (http://www.affymetrix.com/).

### Plant materials and growth conditions

The Egyptian wheat (*Triticum aestivum*) variety, Giza 168 was obtained from the Agricultural Research Centre; ARC, Giza, Egypt. The British variety Oakley was obtained from KWS, UK, Ltd. To test osmotic tolerance, seeds were exposed to 20% (w/v) PEG-6000 as an osmotic-stress inducing medium. Thirty seeds were germinated on filter paper in petri dishes wetted with 7 ml of distilled water or 20% PEG solution using three replicates for each variety and treatment, then the number of germinated seeds was counted to calculate germination percentage (see Supplementary Fig. S1 at *JXB* online). Germination was scored when radicles reached 5mm in length.

To analyse ABA responses of wheat plants seeds were germinated in pots containing compost in a growth chamber at 20 °C, 16 h photoperiod, 60% RH and watered twice per week. ABA (100 µM) was applied as foliar sprays at 9 days after sowing (DAS). Gas exchange parameters were determined for control and ABA-treated plants 24 h following ABA application using a commercial, open-flow gas exchange measurement system (LI-6400P, LI-COR Inc., Lincoln, NE). Biochemical methods were used for measuring osmolyte concentrations one week after ABA treatment as follows: soluble sugars were extracted according to (Schortemeyer *et al.*, 1997) and determined according to (Schlüter and Crawford, 2001), proline was determined according to (Bates *et al.*, 1973), glycine-betaine was determined according to (Grieve and Grattan, 1983) and amino acids concentrations were determined according to (Sircelj *et al.*, 2005).

For moss, WT protonemal tissue was sub-cultured at weekly intervals on cellophane overlays on solid BCD medium containing 5mM diammonium tartrate and trace elements (BCDAT) (Knight *et al.*, 2002). ABA-treated (BCDAT supplemented with 10^−5^ M ABA, 1 h). Tissue was harvested and squeezed dry before freezing in liquid nitrogen and storage at –70 °C before RNA isolation. Protonemal tissue from a line expressing a peroxisomal targeted mRFP (Kamisugi *et al.*, 2016) was sub-cultured on cellophane overlays on solid BCD medium containing 1mM CaCl_2_ 5 mM diammonium tartrate. Seven days old protonemal tissue on the cellophane discs was transferred to petri dishes containing BCDAT with or without 10^−5^ M ABA for 6 h before counting peroxisomes by fluorescence microcopy. The number of peroxisomes per cell was determined for at least 23 randomly selected cells. Analysis of variance (ANOVA) was performed to identify significant differences between the treatments with a level of significance of a *P*≤0.05. For determining the significant effects between the treatments, comparison was made using the least significant difference (LSD) test with a *P*≤0.05.

### RNA extraction and qPCR

About 0.1g wheat leaf tissue was harvested 24h after ABA treatment and homogenized with liquid nitrogen and total RNA was extracted using an RNeasy mini kit (Qiagen) and treated with RNase-Free DNase (Qiagen) following the instruction protocol. For moss samples, RNA was extracted according to (Knight *et al.*, 2002). For rt-PCR, RNA (10 µg) was digested with 1 unit of RQ1 DNase (Promega) for 10 min at room temperature and purified by phenol-chloroform extraction and ethanol precipitation. RNA for all samples was quantified by nanodrop spectrophotometry then the purity and integrity of total RNA was assessed by Agilent BioAnalyzer. Complementary DNA was synthesized from 1 µg of RNA using the BioRad iscript select cDNA synthesis kit. The reaction mixture was diluted 30-fold with water, and 2 µl aliquots were used for PCR amplification. Quantitative real-time polymerase chain reaction (qPCR) was performed with the diluted cDNA samples in a 20 μl reaction mixture containing 10 µl BioRad iQ SYBR 2X mix and 300 nM PCR primers. PCR was performed using a BioRad Cfx Manager as follows: denaturation for 2 m at 95 °C, followed by 40 cycles of 10 s at 95 °C, and 30 s at 60 °C. The PCR amplification efficiency was determined for each primer combination automatically calculated by the BioRad CFX Manager software using the input information of standard concentrations and dilutions used into the program. The standard curve was 5 serial dilutions of a mixture of all sample cDNAs. The PCR efficiencies ranged from 87 to 110%. Three biological replicates and three technical replicates were used for each treatment. No signals were detected in any reaction without template that had been used as a negative control (NTC). The relative transcript levels were calculated using the 2^−∆∆Ct^ method, with the wheat glyceraldehyde3-phosphate dehydrogenase (GAPDH) and moss Clathrin Coat Assembly Protein AP50 (CAP50: Pp3c27_2250V3.1) genes as internal controls. Primer pairs are listed in Supplementary Table S1.

## Results

### Identification of peroxisome associated genes in wheat and moss

Three sets of genes associated with peroxisome biogenesis and function were identified in wheat and moss using the corresponding Arabidopsis proteins as queries. These 3 gene sets coded for i) proteins carrying a predicted peroxisome targeting signal type 1 (PTS1) sequence at the C-terminus (‘genes of putative PTS1 proteins’) ii) enzymes involved in the cellular antioxidant network that had been described as peroxisomal, their non-peroxisomal homologs and some additional non-peroxisomal antioxidant enzymes (‘*Antox* proteins’) and iii) *PEX* genes involved in peroxisome biogenesis. Table 1 summarises the number of genes in these gene sets in Arabidopsis and their corresponding homologs in moss and wheat. In total 340 genes of putative PTS1 proteins from Arabidopsis, identified 1052 homologs in wheat and 282 homologs in moss. Some of these homologs were present in multiple copies and only 185 gene products were predicted to contain PTS1 in wheat and 108 in moss Supplementary Tables S2 and S3 show the identified genes of putative PTS1 proteins in moss and wheat respectively.

**Table 1.** Numbers of genes studied in Arabidopsis included in this study and the numbers of their corresponding homologs in moss and wheat

Gene group	Arabidopsis	Wheat	Moss
***PTS1***	337 predicted as PTS1 3 non-canonical PTS1 (Catalase)^†^	185* (of 65 *A. thaliana PTS1* genes)	108 (of 63 *A. thaliana PTS1* genes)
Total number of homologs identified		1052 (of 85 *A. thaliana PTS1* genes)	282 (of 146 *A. thaliana PTS1* genes)
Number of homologs predicted to contain PTS1		228 (of 64 *A. thaliana PTS1* genes)	100 (of 60 *A. thaliana PTS1* genes)
		3 paralogs for 1 catalase	8 paralogs for 3 catalase
**Antioxidant enzymes**	51	94 (of 27 *A. thaliana* genes)	49 (of 26 *A. thaliana* genes)
**PEX**	22	46	27

*For 46 PTS1 proteins of Arabidopsis the corresponding homologs in wheat could not identified due to major differences in protein sequences in Gramene and phytozome databases as described in the Methods.

^†^Catalase (*CAT*) genes are included in the antioxidant genes set but they also possess a non-canonical PTS1 which although functional deviates from the PTS1 consensus

The 51 ‘Antox’ genes from Arabidopsis identified 94 homologs in wheat and 49 in moss (Table 2). For the Arabidopsis proteins the known/predicted location according to SUBA (the subcellular localization database of Arabidopsis proteins (Hooper *et al.*, 2017) and for the identified moss and wheat homologues the favoured subcellular location predicted using Plant-mPloc (Chou and Shen, 2010) is given in Supplementary Tables S4 and S5 respectively. The *PEX* gene complement of wheat and moss has been described (Cross *et al.*, 2016).

**Table 2.** Antioxidant homologous gene numbers in Arabidopsis, moss and wheat

Gene Name	*A. thaliana*	*P. patens*	*T. aestivum*
Catalase (*CAT*)	3	8	3
Ascorbate Peroxidase (*APX*)	8	8	22
Monodehydroascorbate reductase (*MDAR*)	5	4	12
Glutathione reductase (*GR*)	2	2	9
6-phosphogluconate dehydrogenase	8	7	10
Isocitrate dehydrogenase (*ICDH*)	9	9	17
6-Phosphogluconolactonase	5	3	7
Glutathione peroxidase (*GPX*)	8	3	9
L-ascorbate oxidase	1	1	2
Dehydroascorbate reductase (*DHAR*)	2	4	3
**Total counts**	51	49	94

### Expression of peroxisome related genes in moss in response to ABA, osmotic stress and dehydration

**Fig. 1.**
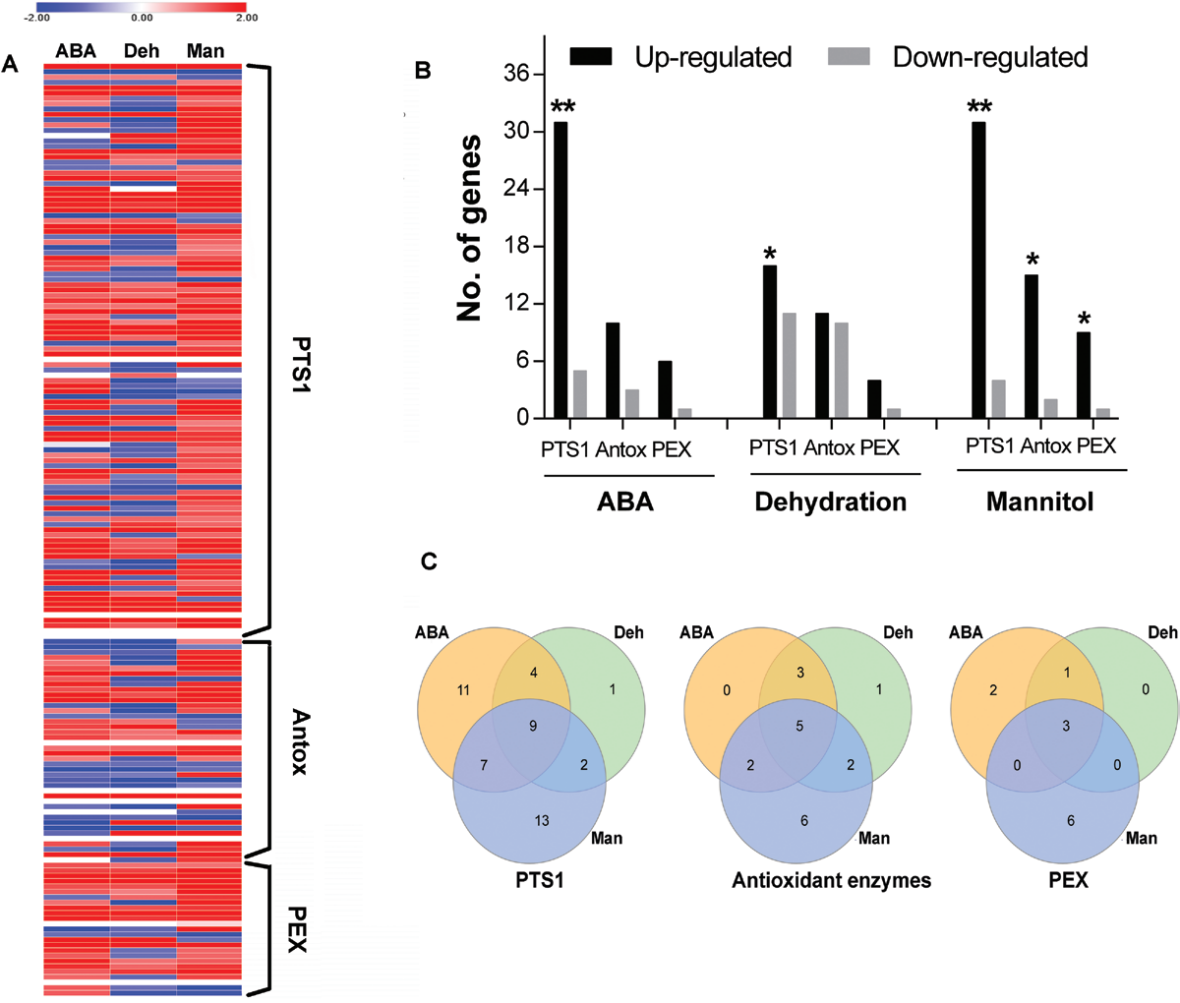
Numbers of differentially regulated genes in *P. patens* in response to 10 µM ABA, 70% dehydration and osmotic stress (10% (w/v) mannitol treatment) conditions. **A:** peroxisomal genes expression under control, ABA, dehydration and mannitol. Red colour indicates a higher expression level and blue colour indicates a lower expression level, white indicates unexpressed gene. Each row is normalized to control. **B:** Total numbers of differentially regulated *PTS1*, *Antox* and *PEX* genes Black bars indicate up-regulated genes, pale grey bars indicate down-regulated genes. **C:** Numbers of genes up-regulated ≥2FC in response to ABA, osmotic stress and dehydration in wild-type protonemal tissue of *Physcomitrella patens*. Star symbol indicates significant hypergeometric probability at *P*≤0.05, two stars for *P* value≤2.3E-02.

In order to investigate the response of peroxisome related pathways under ABA and drought stress conditions in moss we analyzed the changes in expression of the putative *PTS1*, *Antox* and *PEX* genes listed in Supplementary Tables S2 and S4 and (Cross *et al.*, 2016) in the *Physcomitrella* RNAseq datasets. The genes upregulated by >2-fold change for each of these gene sets under conditions of 10 µM ABA treatment, osmotic stress (10% mannitol) or dehydration (70% loss of fresh-weight) are shown in Fig. 1 and gene identification numbers of each set are indicated in Supplementary Table S6. While there are commonalities, each stress has its own distinct signature (Fig. 1A). The numbers of up- and down-regulated genes under the 3 conditions is shown in Fig. 1B. Using hypergeometric probability, statistically significant numbers of genes of putative PTS1 proteins were up-regulated >2FC in response to ABA, dehydration and mannitol compared to the total number of up-regulated genes in the whole dataset under each condition, whereas the number of up-regulated *Antox* genes and *PEX* genes were statistically significant only under osmotic stress by mannitol.

**Fig. 2.**
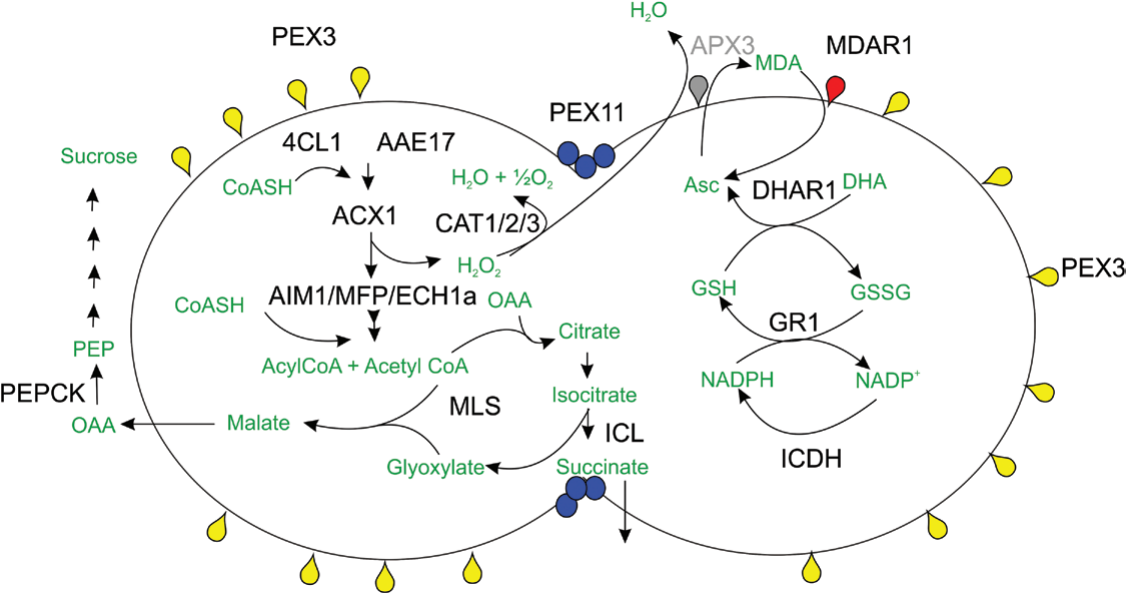
Diagram showing metabolic relationships between selected peroxisomal proteins and enzymes upregulated by drought or ABA. Protein names are shown in black typeface and metabolites in green. 4CL1 and AAE17 activate (unknown) substrates for β-oxidation, which utilizes enzymes acylCoA oxidase (ACX)1 and multifunctional proteins MFP2 and/or AIM1. ECH1a is a poorly characterized member of the enoylCoA hydratase/isomerase family. ACX (and other peroxisomal enzymes) produce hydrogen peroxide that is broken down by catalase (CAT). Acetyl CoA produced can enter the glyoxylate pathway; malate synthase (MLS) and isocitrate lyase (ICL) are the unique enzymes of this pathway which produces malate that is exported as a substrate for gluconeogenesis. Phosphoenolpyruvate carboxykinase (PEPCK) is the first committed step of this pathway, leading to production of sucrose and/or compatible osmolytes. Succinate produced by the ICL reaction is exported to mitochondria for further metabolism. In Arabidopsis, citrate can be exported directly for respiration (not shown). On the right hand side of the diagram a simplified representation of the ascorbate glutathione cycle is shown. Asc; ascorbate; DHA dehydroascorbate; GSH reduced glutathione; GSSG; oxidized glutathione. DHAR dehydroascorbate reductase. GR1 glutathione reductase; ICDH NADP+ dependent isocitrate dehydrogenase. Ascorbate peroxidase (APX) and monodehydroascorbate reductase (MDAR) are membrane bound proteins that participate in removal of hydrogen peroxide and regeneration of ascorbate. PEX3 is involved in import of peroxisome membrane proteins (including the components of the import machinery for matrix proteins) and PEX11 is involved in peroxisome division.

Nine genes of putative PTS1 proteins were upregulated under all 3 conditions (Fig. 1C). These were two copper amine oxidases (Pp3c14_14330 and Pp3c17_5710), two acyl adenylate activating enzymes (*AAE17* and *4CL1*; Pp3c1_12140 and Pp3c26_11730 respectively), an *AIM1* homologue which is a multifunctional protein of the β-oxidation pathway (Pp3c1_580) and *ECH1a* which is an enoyl CoA hydratase also potentially associated with β-oxidation (Pp3c23_900). Two isoforms of isocitrate lyase (*ICL*; Pp3c7_2440and Pp3c7_2470), and one isoform of malate synthase (*MS*; Pp3c20_22510) the unique enzymes of the glyoxylate cycle which convert acetyl CoA into malate for gluconeogenesis were also upregulated. *Antox* genes upregulated (Fig. 1C) were two catalase isoforms (Pp3c18_13590 and Pp3c19_6540) and isocitrate dehydrogenase (*ICDH*; Pp3c20_22810). It was noteworthy that this isoform is predicted to contain a PTS1 motif –SKL. Two enzymes associated with the ascorbate-glutathione cycle; dehydroascorbate reductase (*DHAR2*; Pp3c15_21480), glutathione reductase (*GR2*; Pp3c4_17890), probably plastid isoforms, were also upregulated. Two *PEX3* genes (Pp3c8_16550 and Pp3c20_5170) and one *PEX11* gene (PEX11-6, Pp3c3_15780) were upregulated under all treatments (Fig. 1C). The metabolic interrelationships of the proteins encoded by these genes is shown in Fig. 2.

**Fig. 3.**
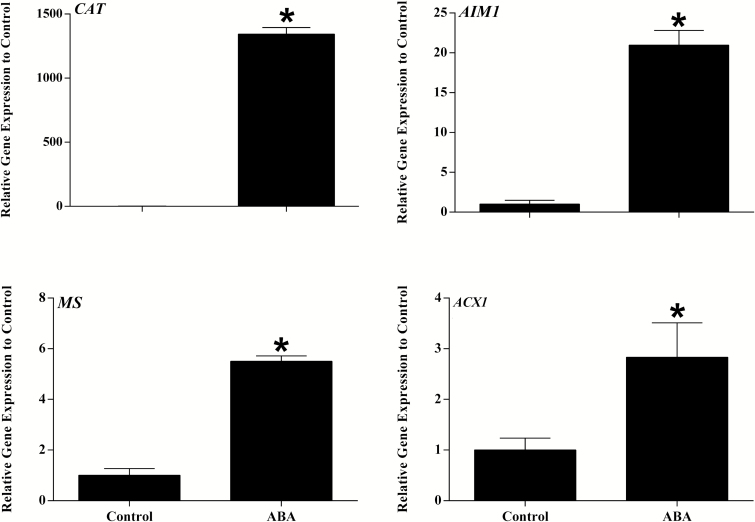
Induction of marker genes for β- oxidation and glyoxylate cycle by ABA treatment in *P. patens*. Quantitative gene expression for catalase (*CAT*), Pp3c19_6540; abnormal inflorescence meristem 1, fatty acid multifunctional protein (*AIM1*), Pp3c1_580; Malate synthase (*MS*), Pp3c20_22510; Acyl-CoA oxidase Pp3c14_14860. Data are expressed as fold change in expression (y-axis) relative to ABA untreated control. Three biological replicates and three technical replicates were used for each treatment. Bars represent the standard error. *Data are significant at *P≤*0.05.

To validate the effect of ABA treatment on gene expression, rt-QPCR was carried out. Catalase was upregulated more than three orders of magnitude upon ABA treatment (Fig. 3). Acyl CoA oxidase 1 (*ACX1*) and *AIM1*, markers for the β-oxidation pathway, were upregulated ~3-fold and >20-fold respectively. Malate synthase, marker for the glyoxylate cycle was upregulated >5-fold by ABA treatment (Fig. 3).

### Induction of peroxisome biogenesis in *Physcomitrella patens* by ABA

**Fig. 4.**
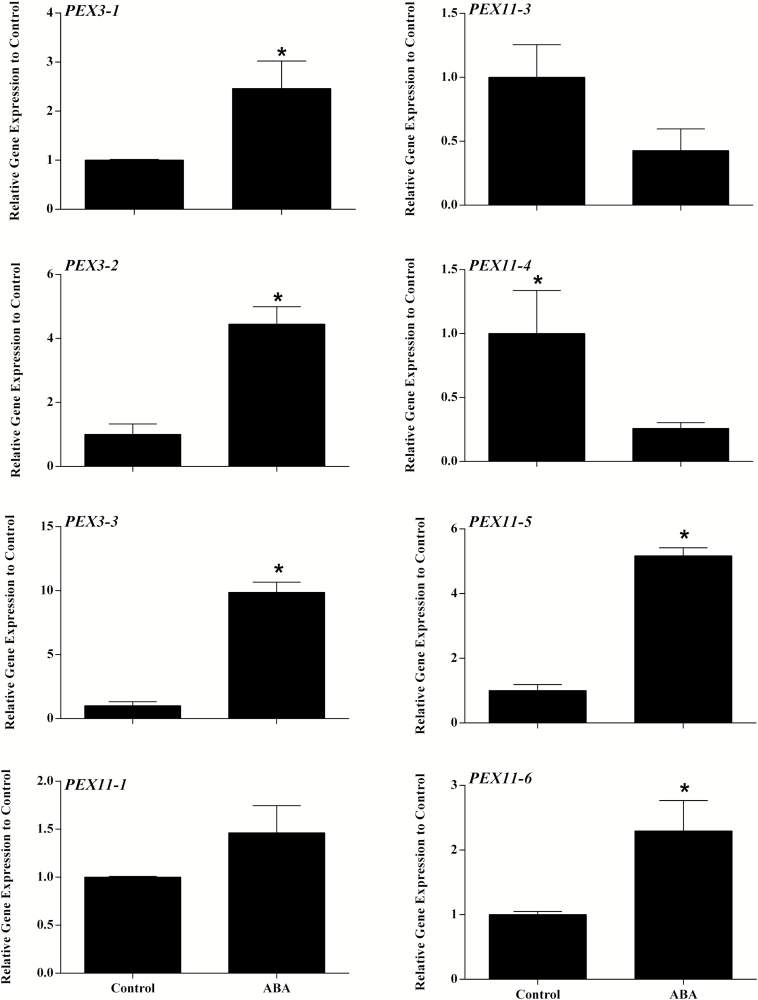
Quantitative real time-PCR analysis of *PEX3* and *PEX11* gene families in *P. patens* in response to ABA. *PEX3-1*, Pp3c24_12050; *PEX3-2*, Pp3c8_16550; *PEX3-3*, Pp3c20_5170; *PEX11-1*, Pp3c19_20730V3.1; *PEX11-3*, Pp3c19_20730; *PEX11-4*, Pp3c24_12360; *PEX11-5*, Pp3c2_11370; *PEX11-6*, Pp3c3_15780. Data are expressed as fold change in expression (y-axis) relative to ABA untreated control. Three biological replicates were used and three technical replicates for each treatment. Bars represent the standard error. *Data are significant at *P*≤0.05.

**Fig. 5.**
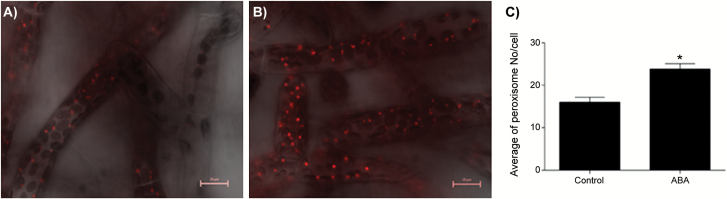
Increased peroxisome biogenesis by ABA in *P. patens* chloronemata. **A**: control, **B**: ABA-treated, **C**: peroxisome number±SE, 23 cells were used to count the peroxisomes and analysis of variance (ANOVA) with a level of significance of a *P*≤0.05 to identify significant differences between the treatments. For determining the significant effects between the treatments, comparison was made using the least significant difference (LSD) test with a *P*≤0.05.

Fig. 4 shows expression of the 3 members of the *PEX3* gene family and 5 members of the *PEX11* family in response to ABA treatment. *PEX3-3* was the most highly induced showing ~10-fold increase in transcript level although both *PEX3-1* and *PEX3-2* showed an increase in transcript level in response to ABA. The *PEX11* family members showed strikingly different responses to ABA. PEX11-1 was unresponsive, *PEX11-3* and *PEX11-4* are down-regulated whilst *PEX11-5* and *PEX11-6* were upregulated ~5 and ~2.5-fold respectively, consistent with the RNA-seq data. As PEX3 and PEX11 both have roles in peroxisome proliferation/division, peroxisomes were counted in *P. patens* chloronemata expressing a peroxisomal targeted RFP that had been ABA-treated for 6 hours (Fig. 5A, B). The mean peroxisome number per cell in ABA-treated samples was 24 compared to 16 in untreated cells which was significant at the *P*=0.05 level (Fig. 5C).

### Expression of peroxisome related genes in *Arabidopsis thaliana* in response to ABA

For comparison with the ABA responses seen in moss, RNAseq data for ABA-treated 10-day old Arabidopsis seedlings was examined. The results for *PTS1*, antioxidant and *PEX* gene sets are summarised in Supplementary Tables S7, S8 and S9 respectively). Genes which showed upregulation by all 3 treatments in moss (see Supplementary Table S6 at *JXB* online) and which are also upregulated >2FC by ABA in Arabidopsis are listed in Table 3. These include two adenylate activating enzymes (*AAE17;* At5g23050 and *AAE12*; At1g65890) and the β-oxidation enzyme *ACX1* (At4g16760). A glutathione reductase *(GR1* At3g24170) and a dehydroascorbate reductase (*DHAR1* At1g19570) associated with the peroxisomal ascorbate-glutathione cycle also showed up-regulation of >2FC. However, unlike moss, glyoxylate cycle genes (*ICL* and *MS*) were not upregulated by ABA in Arabidopsis (Supplementary Table S7 and Table 3).Of the *PEX11* family only *PEX11d* (At3g61070) showed up-regulation >2FC under ABA treatment.

**Table 3.** Common up-regulated genes in moss, Arabidopsis and wheat. Moss genes up-regulated by ABA, dehydration and mannitol >2FC. Expression of these genes in Arabidopsis by ABA (Weng et al., 2016) or in wheat by drought (Kadam et al., 2012)

Moss genes up-reg >2FC (ABA, Man & deh)	*A. thaliana* (ABA)	*T. aestivum* (drought)	
		Tolerant	sensitive
**Genes of putative PTS1 proteins:**			
Copper amine oxidase	x	X	x
Acyl activating enzyme, *AAE*	AAE17	AAE7	AAE7
			AAE10
	AAE12		
*4-Coumarate:CoA ligase, 4CL1*	x	ND	ND
Isocitrate lyase, *ICL*	x	√	√
Malate synthase, *MS*	x	√	√
Abnormal inflorescence meristem 1, fatty acid multifunctional protein, *AIM1*	x	ND	ND
Enoyl-CoA hydratase like protein a*, ECHIa*	x	x	√
Acyl-CoA oxidase 1, *ACX1*	√	ND	ND
**Enzymes involved in the cellular antioxidant network**			
Catalase, *CAT*	x	√	√
Glutathione reductase, *GR*	√	ND	ND
Isocitrate dehydrogenase, *ICDH*	x	x	x
Dehydroascorbate reductase, *DHAR2*	√	x	x
**PEX genes**			
*PEX3*	x	x	x
*PEX11*	*PEX11D*	*PEX11D*	*PEX11D PEX11A*

ND = not detected. ND means that this gene is not found in the array or seq-data.

√ and X means shares up-regulation (≥2) with moss or not, respectively.

*AAE17*, 12, 7 and 10 and *PEX11D* and E: are *AAE* and *PEX* family members respectively, expressed in Arabidopsis and wheat.

### Expression of peroxisome related genes in *Triticum aestivum* in drought tolerant and sensitive genotypes under drought stress

**Fig. 6.**
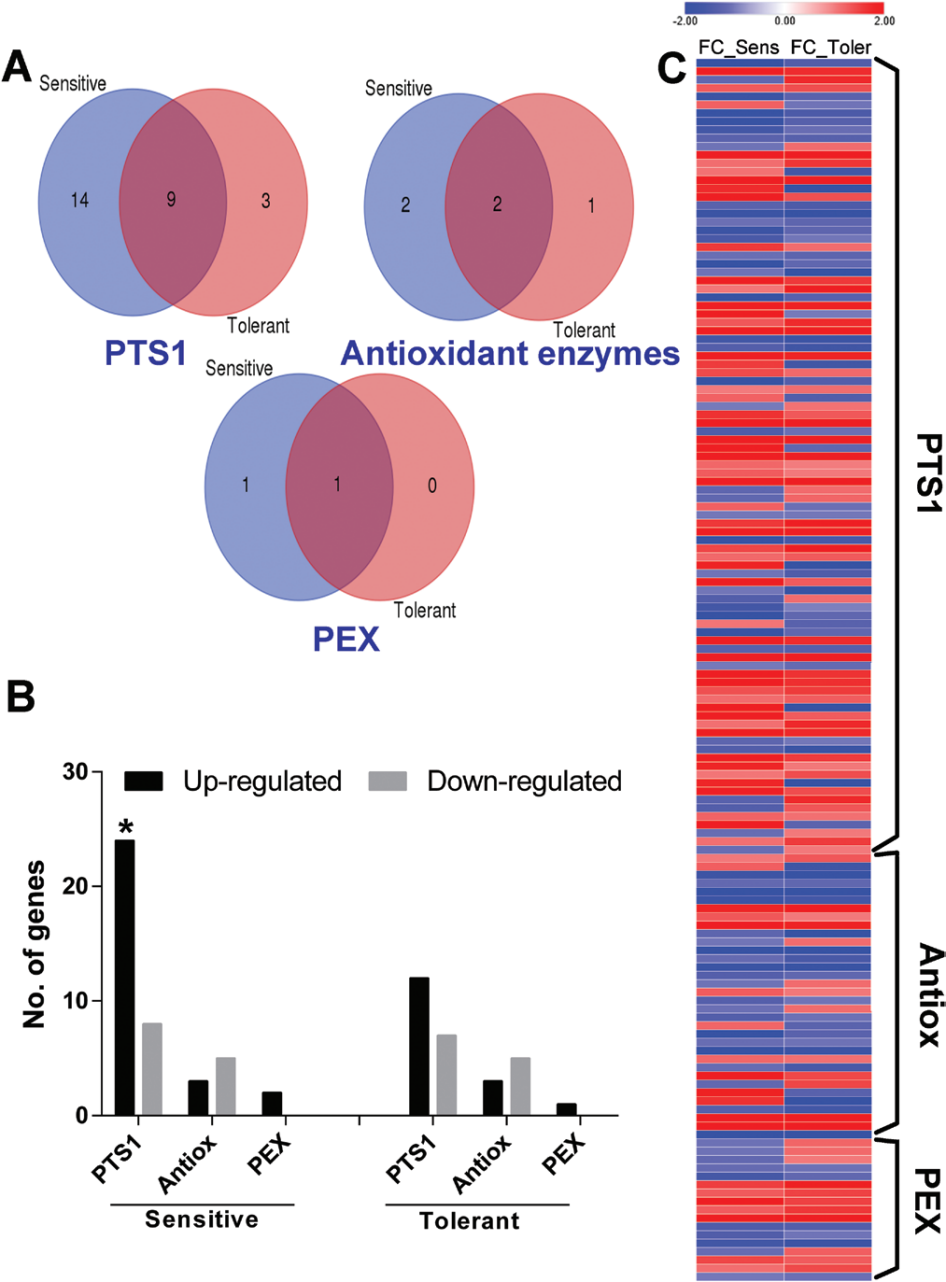
Differentially regulated peroxisomal genes in wheat in response to drought stress in sensitive and tolerant cultivars in GEO dataset GSE30436. **A:** Numbers of *PTS1*, *Antox* and *PEX* genes up-regulated ≥2FC in response to drought stress in sensitive and tolerant wheat cultivars. **B:** Total numbers of differentially regulated genes of *PTS1*, *Antox* and *PEX* genes. Black bars indicate up-regulated genes, pale grey bars indicate down-regulated genes. Star symbol indicates significant hypergeometric probability at *P*≤0.01). **C:** Peroxisomal gene expression under drought stress in sensitive and tolerant cultivars. Blue colour indicates a lower expression level and red colour indicates a higher expression level. Each row normalized to control.

To extend the comparison of peroxisomal responses to wheat, homologs for PTS1, antioxidant enzymes and PEX proteins were identified using the corresponding Affymetrix probe ID (see Supplementary Tables S10, S11 and S12 at *JXB* online). A microarray data set of two sets of bulked recombinant inbred lines which differed in their drought tolerance (Kadam *et al.*, 2012) were analysed for differences in expression of these 3 sets of genes. The values of FC calculated for treated samples compared to control samples are presented Fig. 6. Of the upregulated genes of putative PTS1 proteins 9 are shared, 3 are unique to the tolerant and 14 unique to the sensitive genotype (Fig. 6A). A total of 23 genes of putative PTS1 proteins were significantly up-regulated ≥2FC in the drought sensitive genotype with hypergeometric probability of 0.01 and 8 genes were down-regulated (Fig. 6B). In the drought tolerant genotype 12 genes of putative PTS1 proteins were up-regulated and 7 were down-regulated. It was notable that *ICL* and *MS* and *AAE* were commonly up-regulated in sensitive and tolerant genotypes but with a higher FC in the sensitive genotype although the tolerant genotype had a higher level of expression under the control condition (Supplementary Table S10 and Table 3). Out of 34 Affymetrix wheat probes for *Antox* genes (Supplementary Table S11), 3 genes were up-regulated in the tolerant samples (Fig. 6A). *APX5* and *CAT* were the common up-regulated *Antox* genes under drought conditions in both cultivars. The wheat array contained only 17 probes for *PEX* genes (Supplementary Table S12). Only *PEX11d* was up-regulated ≥2FC in the drought tolerant wheat while *PEX11a* and *PEX11d* were upregulated in the sensitive cultivar. No *PEX* genes were down-regulated ≥2FC in either cultivar (Fig. 6B). Table 3 shows the genes in wheat that are upregulated by drought where homologues are also up-regulated by ABA in Arabidopsis and by ABA, dehydration and mannitol in moss.

### Gene expression of peroxisome related genes in drought tolerant and sensitive cultivars of wheat in response to ABA

**Fig. 7.**
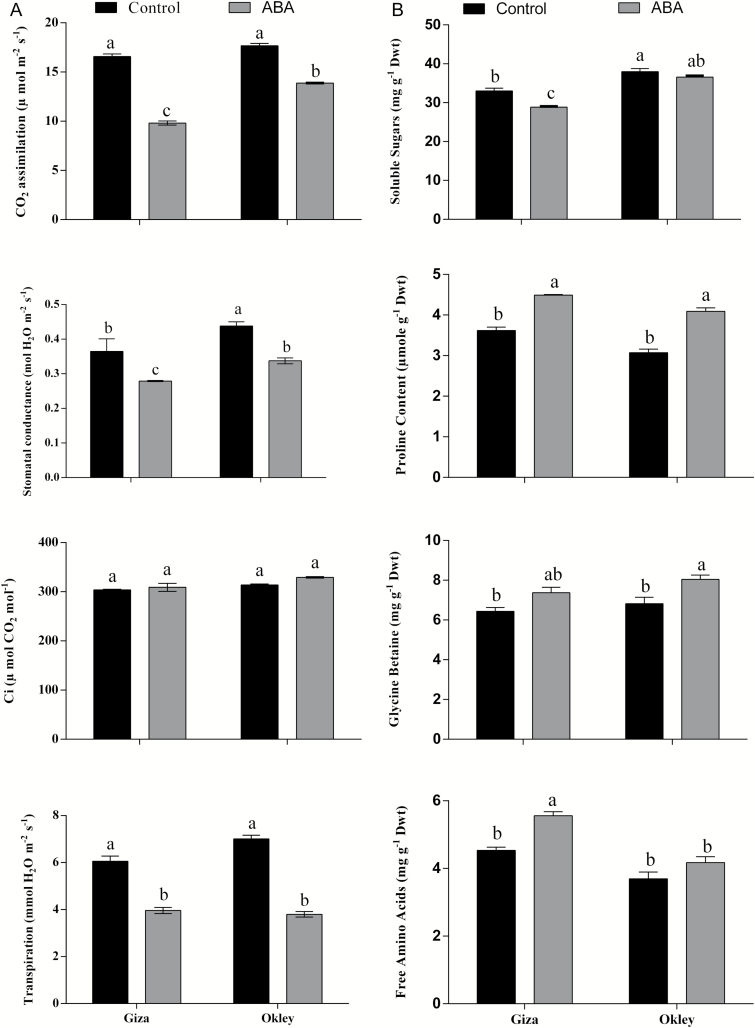
Physiological and biochemical performance of two wheat cultivars Giza and Oakley in response to ABA treatment. **A:** Photosynthesis parameters in 10 days old seedlings after 24 hours of 100µM ABA treatment. **B:** Osmoprotectant compounds analysis in two wheat cultivars Giza and Oakley after 7 days of 100 µM ABA treatment. Data are means of 3 replicates±SE. Bars labelled with different letters are significantly different at *P*≤0.05.

To further explore the relationship between expression of peroxisome related genes in response to drought tolerance, two wheat genotypes differing in their performance under drought stress were selected based on a germination tolerance test using PEG-6000 and calculating water content percentage (see Supplementary Fig. S1 at *JXB* online). These data suggest Giza is less tolerant to drought than Oakley. Carbon dioxide assimilation was measured in both cultivars with and without 100 µM ABA treatment after 24 hours (Fig. 7A). In both cultivars, CO_2_ assimilation decreased as a result of closure of stomata by ABA, and this is reflected by the decrease in stomatal conductance and transpiration in both cultivars. However, both cultivars maintained stable levels of internal carbon. Giza, the more sensitive variety, is the most affected in terms of CO_2_ assimilation (Fig. 7A). Osmolyte accumulation after 7 days in both cultivars showed variation (Fig. 7B). Soluble sugars significantly decreased in Giza upon ABA treatment, presumably as a result of decreased photosynthesis. However, in Oakley the soluble sugars did not significantly change compared to the untreated control. Proline and glycine betaine accumulated in both cultivars in response to ABA. Free amino acids were elevated in response to ABA in Giza which may reflect proteolysis; however this response was much less marked in Oakley (Fig. 7B).

**Fig. 8.**
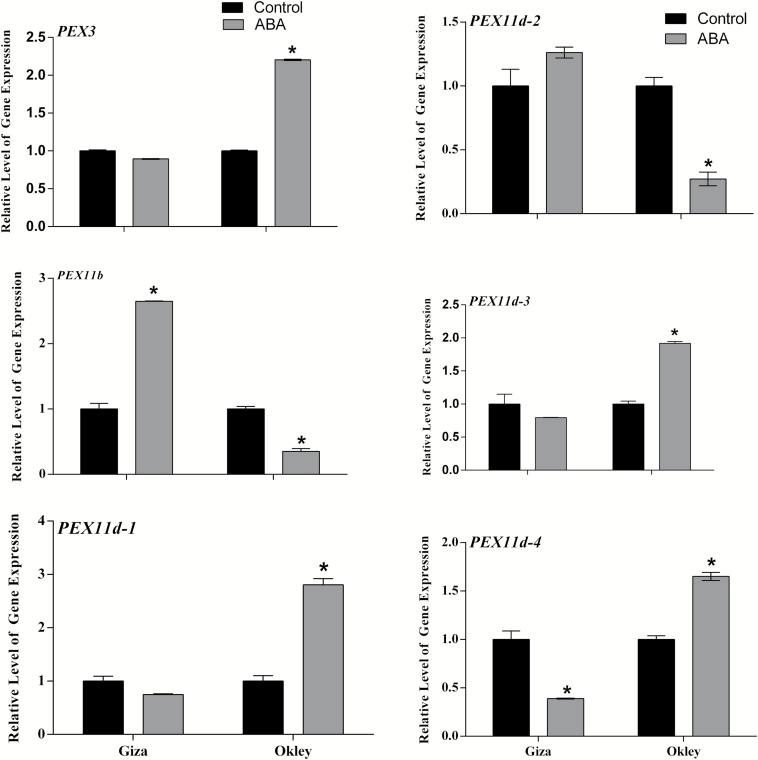
Quantitative real time-PCR analysis of *PEX3* and *PEX11* in two cultivars of *T. aestivum*, Giza168 and Oakley in response to 100 µM ABA after 24 hours. Data are expressed as fold change in expression (y-axis) relative to ABA untreated control. Three biological replicates were used and three technical replicates for each treatment. Bars represent the standard error. Star symbols indicate significance of t-test for control and treated samples. *Data are significant at *P*≤0.05.

Expression of *PEX3* and the *PEX11* family was studied in these leaf samples 24 hours after ABA treatment by rtQPCR (Fig. 8). *PEX3* (the conserved region in 3 *PEX3* genes; Traes_5DS_BB388ED7C, Traes_5AS_6B30155C7 and Traes_5BS_8560EC011) was strongly upregulated by ABA treatment in the tolerant variety Oakley but not in the sensitive cultivar Giza. Three *PEX11d* isoforms d-1; Traes_4AL_D9FFAAA1A, d-3; Traes_7AS_D51B7852F and 4; Traes_7DS_DA11E7020 were also upregulated in Oakley but not Giza. Giza showed significant up-regulation of *PEX11b*; Traes_2AL_FB2B6601D and slight increase in *PEX11d-2; Traes_4BL_DD7569D22*.

## Discussion

This study has collated an inventory of predicted peroxisomal PTS1 targeted proteins (genes of putative PTS1 proteins) from moss and wheat. It has also collated information on the network of anti-oxidant enzymes (*Antox* genes) from these species, both probable peroxisome isoforms and their non-peroxisomal homologues. Using this information, along with previously collated information on the *PEX* gene complement of these species we have examined transcriptional responses of these gene sets to drought, dehydration and ABA response with the intention of identifying evolutionary conserved responses

### The peroxisome proteome of moss and wheat

The results summarized in Table 1 show that not all PTS1 proteins in Arabidopsis have obvious homologues in wheat and moss, whilst among the homologous proteins identified in these two species, some appear to lack a PTS1. In some cases this could be due to a false positive prediction of peroxisome targeting of the corresponding Arabidopsis protein, as not all have been experimentally validated. Also possible is potential false negative predictions arising from yet unknown variation in PTS1 usage in moss and wheat, and the potential for ‘piggy back import’ (Kataya *et al.*, 2015) where a protein lacking a PTS1 can be co-imported with a partner that has a PTS1. Nevertheless despite these caveats the data suggest that the peroxisome proteome is quite variable between organisms. This fits with the proposal that peroxisome targeting by the PTS1 pathway can evolve relatively rapidly through alternative splicing, point mutation and stop codon read through (Reumann *et al.*, 2016). The overrepresentation of *PTS1* gene transcripts amongst those upregulated across species points to the importance of peroxisome processes in response to drought and ABA.

### Evidence for conserved upregulation of peroxisomal β-oxidation

Members of the acyl adenylate activating family of enzymes were upregulated in all 3 species. These enzymes activate diverse substrates for entry into β-oxidation. *AAE17* in Arabidopsis is the closest relative of *AAE18* which activates the synthetic pro hormone 2,4-dichlorophenoxy butyric acid (2,4DB) for β-oxidation but the natural substrates are not known for either enzyme (Wiszniewski *et al.*, 2009). rtQPCR confirmed significant induction of *ACX1* and *AIM1* by ABA treatment in moss (Fig. 3). *ACX1* is also induced by ABA in Arabidopsis (see Supplementary Table S7 and Table 3 at *JXB* online) and by wounding, dehydration and Jasmonic acid (JA) (Castillo *et al.*, 2004).

Jasmonates have been reported to interact with ABA signaling in drought stress (see (Riemann *et al.*, 2015) for recent review). As drought is proposed to lead to a block in OPDA conversion to JA, and OPDA is an important signal for guard cell closure which regulates guard cell aperture both co-operatively with, and independently of, ABA (Savchenko *et al.*, 2014), it therefore seems unlikely that ABA increases JA production via upregulation of peroxisomal β-oxidation. Neither *OPR3* or *OPCL1*, which are key enzymes in the pathway, change in Arabidopsis on ABA treatment (see Supplementary Table S7 at *JXB* online). *Physcomitrella patens* is reported to contain cyclopentanones (OPDA) but not jasmonates (Stumpe *et al.*, 2010). Why then should peroxisomal β-oxidation be upregulated? Stress responses trigger lipid dependent signaling (Hou *et al.*, 2016) and peroxisomal β-oxidation could be involved in turnover of some of these molecules or in degradation of peroxidated membrane lipids formed as a result of oxidative stress. Metabolism of the yet unknown substrates activated by the AAEs may also be important signals or mitigators of stress responses.

### The glyoxylate cycle—upregulation for production of carbohydrates?

Fatty acid breakdown produces acetyl CoA which can enter the glyoxylate cycle (Eastmond *et al.*, 2000; Eastmond and Graham, 2001). Induction of isocitrate lyase and malate synthase, provides a route for synthesis of malate which can be converted to oxaloacetate, the starting point for gluconeogenesis (Fig. 2). Consistent with this hypothesis, the moss gene Pp3c4_25090 encoding a PEP carboxykinase (the first committed step of gluconeogenesis) was upregulated 3.2, 4.4 and 2.7-fold under ABA, mannitol and dehydration treatment. Gluconeogenesis can provide soluble sugars for respiration or osmotic balance under water deficit conditions, and sucrose accumulates to high levels in *P. patens* following ABA treatment (Oldenhof *et al.*, 2006). Strikingly, the glyoxylate cycle enzymes were not induced upon drought or ABA treatment in Arabidopsis ((Li and Hu, 2015) and see Supplementary Table S7 at *JXB* online). Non coordinate induction of β-oxidation and glyoxylate cycle was seen during starvation or senescence in Arabidopsis (Charlton *et al.*, 2005*a*) supporting the notion that lipid is broken down and respired (Pracharoenwattana *et al.*, 2005) in contrast to other species where it feeds into the glyoxylate cycle.

### Branched chain amino acid metabolism

Another source of substrates for β-oxidation in non-lipid storing tissue is degradation of branched chain amino acids (BCAAs). The expression of *BCAT5*—the first key enzyme in degrading BCAAs—and *IVDH* (Isovaleryl-CoA-Dehydrogenase), the enzyme that converts acyl CoA to enoyl CoA (two copies of *BCAT5* and two copies of *IVDH* in moss), both show strong upregulation under ABA, dehydration and mannitol treatment, as does *ECH1a*, an enoylCoA dehydratase. In Arabidopsis the pathway for degradation of BCAAs is predominantly mitochondrial (Binder, 2010) but at least one step of valine degradation catalyzed by hydroxyisobutryl CoA hydrolase (encoded by the *CHY1* gene) is peroxisomal (Zolman *et al.*, 2001). Intriguingly *chy1* mutants are defective in cold responses, are more sensitive to dark induced damage and accumulate ROS; phenotypes which can be suppressed by exogenous application of sucrose suggesting a role in osmoprotection and/or maintenance of carbohydrate levels (Dong *et al.*, 2009).

### A role for peroxisomes in stomatal movement

An early response to water stress is stomatal closure and recent data points to a role for peroxisome metabolism in guard cells in regulating stomatal movement. Peroxisomal β-oxidation of stored lipids contributes to ATP production for stomatal opening in both Arabidopsis and the lycophyte *Selaginella* (McLachlan *et al.*, 2016). Furthermore, an Arabidopsis mutant defective in peroxisomal NADP+ dependent isocitrate dehydrogenase showed deficiency in stomatal opening which was rescued by ascorbate. This led to the proposal that loss of peroxisomal NADP^+^ dependent ICDH activity impacts the ascorbate glutathione cycle leading to increased cytosolic H_2_O_2_ (Leterrier *et al.*, 2016). An ICDH isoform (Pp3c20_22810) with a putative PTS1 sequence was upregulated by all treatments in moss.

### Peroxisomes and ROS

Increased photorespiration as a result of stomatal closure under water deficit leads to increased production of hydrogen peroxide as a consequence of increased photorespiratory flux. Interestingly though, none of the candidate glycolate oxidases were upregulated >2-fold. Antioxidant responses are complex and sometimes contradictory (Noctor *et al.*, 2014). One significant complication is the likely compartment-specific production of ROS and antioxidants; spatial information which is lost upon biochemical extraction. In the current study, focusing on the response of likely peroxisomal targeted enzymes provides an alternative approach to looking at a compartment specific response. Considering the genes commonly upregulated across the 3 stress treatments in moss, Acyl CoA oxidase and the copper amine oxidases generate H_2_O_2._ Copper-containing amine oxidases (CuAOs) are involved in oxidative de-amination of polyamines, ubiquitous polycationic compounds involved in crucial events in the life of the cell (Tavladoraki *et al.*, 2012). Two catalase isoforms and virtually the complete ascorbate glutathione cycle was upregulated. Glutathione reductase 1 in Arabidopsis is dual targeted to the cytosol and peroxisomes (Kataya and Reumann, 2010), and a peroxisomal isoform of NADP^+^ dependent isocitrate dehydrogenase contributes to the ascorbate glutathione cycle in peroxisomes (Jimenez *et al.*, 1997; Reumann *et al.*, 2007). Measurement of changes in organelle redox using roGFP showed drought primarily affected chloroplast and mitochondrial redox potential whereas peroxisome redox potential was more affected in the dark and was exacerbated by 3-amino triazole treatment which inhibits catalase (Bratt *et al.*, 2016). This suggests an effective peroxisomal antioxidant defence under drought conditions. Upregulation of catalase was seen in moss and wheat under all conditions whilst upregulation of *ACX1*, *GR* and *DHAR2* showed common upregulation between moss and Arabidopsis (Table 3). Wheat (Ford *et al.*, 2011) and moss (Cui *et al.*, 2012; Wang *et al.*, 2009) proteomic studies also support upregulation of antioxidants and polyamine biosynthesis (Cheng *et al.*, 2015) under drought stress.

### Peroxisome biogenesis

Peroxisomes multiply by division of preexisting peroxisomes in a process requiring the PEX11 family of membrane proteins and may also form *de novo* from the ER in a pathway that requires PEX3. PEX3 in addition recruits peroxisomal membrane proteins including membrane bound enzymes and components of the import machinery for peroxisome matrix proteins All three *PEX3* genes in moss were upregulated by ABA treatment, and different members of the *PEX11* gene family were differentially expressed suggesting specialization of function. Consistent with this, ABA triggered peroxisome proliferation in protonemal tissue (Fig. 5).

In Arabidopsis *PEX11b*, *c* and *d* are upregulated under hypoxia and biotic stress and only *PEX11b* and *PEX11d* are upregulated by ABA whereas *PEX11c* is down regulated (Li and Hu, 2015). *PEX11e* induction in response to salt stress requires components of the ABA signaling pathway (Charlton *et al.*, 2005*b*). Salt stress, like drought, imposes a dehydration stress but also an ionic stress and triggers peroxisome proliferation (Mitsuya *et al.*, 2010). Arabidopsis *PEX11b* stimulates peroxisome proliferation in response to light (Desai and Hu, 2008). High light, drought, salt and ABA all trigger ROS production which transcriptionally activates some *PEX* genes (Lopez-Huertas *et al.*, 2000). Exogenous application leads to formation of peroxules, and *PEX11a* is involved in this process (Rodríguez-Serrano *et al.*, 2016). In wheat, striking differences were seen between drought sensitive cultivar Giza and drought tolerant cultivar Oakley with respect to *PEX* gene expression. *PEX3* and *PEX11d1, d3* and *d4* were strongly upregulated by ABA in the resistant cultivar but not in the sensitive one. Conversely *PEX11b* was upregulated in the sensitive cultivar (Fig. 8).

## Conclusions

Peroxisome biogenesis and genes of putative PTS1 proteins are upregulated in response to drought, dehydration and ABA across evolutionary distant plant species. While the specifics of the responses differ, core pathways of PEX3/11 and β-oxidation are conserved. This suggests an important and evolutionarily ancient role for peroxisomes in stress perception and response. As differential regulation of *PEX3* and *PEX11* family members is correlated with better drought tolerance, the accumulation of multiple gene copies has perhaps allowed elaboration in the control of peroxisomal biogenesis in response to stress. Collectively our findings give new insights into the role of peroxisomes and peroxisome associated processes in response to drought and ABA across a wide evolutionary distance and suggest that the role of peroxisomes in perceiving and responding to drought stress is worthy of further investigation.

## Supplementary Data

Supplementary data are available at *JXB* online.

Supplementary Fig. S1. Germination % of two wheat varieties, Giza 168 and Oakley under 20% PEG-6000.

Supplementary Table S1. Primer sequences used in this study.

Supplementary Table S2. List of *Physcomitrella patens* homologues of proven and predicted Arabidopsis PTS1 proteins.

Supplementary Table S3. List of wheat homologues of proven and predicted Arabidopsis PTS1proteins.

Supplementary Table S4. List of Arabidopsis antioxidant gene homologues in *Physcomitrella patens*.

Supplementary Table S5. List of Arabidopsis antioxidant gene homologues in wheat.

Supplementary Table S6. List of *P.patens* differentially regulated genes under ABA, mannitol and dehydration.

Supplementary Table S7. Microarray-based expression data for Arabidopsis genes of putative PTS1 proteins under ABA treatment, GSE65739.

Supplementary Table S8. Microarray based-expression data for Arabidopsis antioxidant genes under ABA treatment, GSE65739.

Supplementary Table S9. Microarray based-expression data for Arabidopsis *PEX* genes under ABA treatment, GSE65739.

Supplementary Table S10. Microarray based-expression data for wheat genes of putative PTS1 proteins under drought, GSE30436.

Supplementary Table S11. Microarray based-expression data for wheat antioxidant genes under drought, GSE30436.

Supplementary Table S12. Microarray based-expression data for wheat *PEX* genes under drought, GSE30436.

## Author contributions

H.T.E.: Designed and performed wheat experiments and moss peroxisome proliferation experiment, identified peroxisomal homologs of moss and wheat, analysed Arabidopsis and wheat RNA-seq and microarray data, and performed real-time QPCR. S.S.: Provided moss cDNA samples, provided help in the bioinformatic analysis and performed some analysis of moss RNA-seq data. A.C.C.: Provided reagents and training for the moss work, designed and supervised moss work. A.B.: Conceived the project with H.T.E. and A.C.C., designed, supervised and coordinated the study and prepared the paper with H.T.E. All authors saw and approved the final version of the manuscript.

## Supplementary Material

Supplementary Figure S1

Supplementary Tables
