# Supplementary material for: Conserved and differential transcriptional responses of peroxisome associated pathways to drought, dehydration and ABA
Source: J Exp Bot. 2018 Jul 19;69(20):4971–85. doi: 10.1093/jxb/ery266 (PMC6137984; doi:10.1093/jxb/ery266)
Supplement: Supplementary Figure S1 [file ery266_suppl_supplementary_figure_s1.pdf]

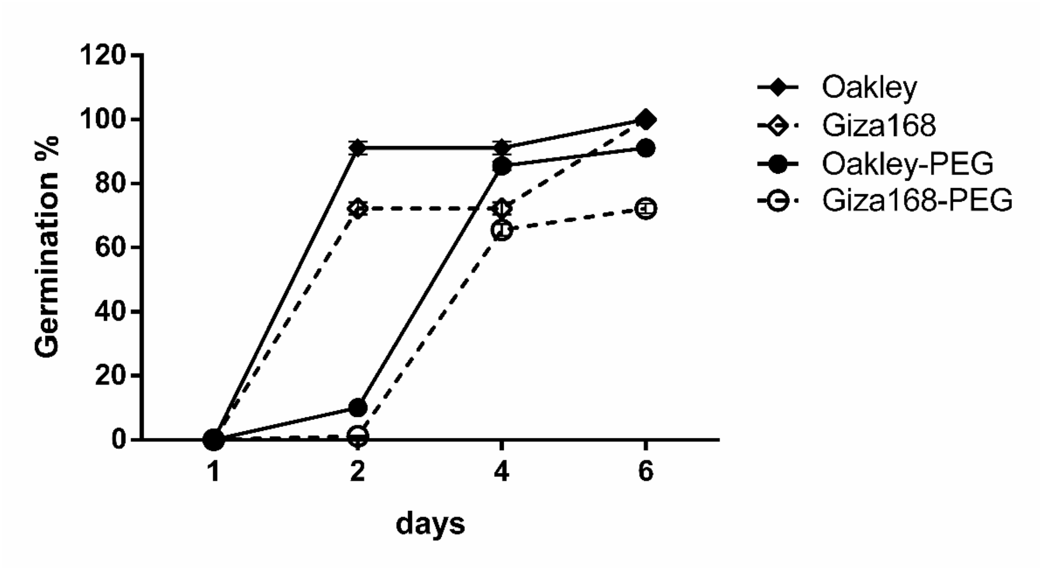

Supplementary Figure 1: Germination % of two wheat varieties, Giza 168 and Oakley under 20% PEG-6000. Data are shown as means ± SE of three independent measurements, 30 seeds have been used to calculate the germination % and analysis of variance (ANOVA) with a level of significance of a $P \leq 0.05$ to identify significant differences between the treatments. For determining the significant effects between the treatments, comparison was made using the least significant difference (LSD) test with a $P \leq 0.05$
